# Non-therapeutic play to overcome negative emotional symptoms and improve emotional intelligence in children aged 3–7: a systematic review

**DOI:** 10.3389/fpsyg.2025.1475387

**Published:** 2025-03-06

**Authors:** Alexander N. Veraksa, Valeriya A. Plotnikova, Vera L. Sukhikh, Dmitry S. Kornienko, Natalia A. Rudnova

**Affiliations:** Laboratory of Childhood Psychology and Digital Socialization, Federal Scientific Center of Psychological and Multidisciplinary Research, Moscow, Russia

**Keywords:** early childhood, cultural-historical approach, psychological help, pretend play, digital play, emotional regulation, anxiety

## Abstract

Play therapy is an effective approach to reduce emotional symptoms, stress and develop emotional sphere in early childhood. But the organization of therapy requires long-term specialized training and a significant number of sessions, making it inaccessible in public institutions. This systematic review aims to analyze the potential and mechanisms of play outside of the therapeutic setting for overcoming emotional symptoms, develop emotional regulation and resilience in preschool-aged children. Using the PRISMA method, we selected 33 articles for qualitative analysis from the 9,639 published articles over the past 5 years found through PubMed, ScienceDirect, and Scopus. The effectiveness of pretend play, play with rules, digital play, and outdoor play for addressing emotional symptoms and improving mental health was revealed. It was shown that play outside of the therapeutic setting is widely used to improve emotional intelligence and its components, decrease anxiety, aggression, and fear. In accordance with the cultural-historical approach, seven generalized blocks of play mechanisms were identified to enhance children’s mental well-being and promote development. Pretend play was found to have the greatest corrective and developmental potential, due to the diverse mechanisms involved. The findings of this review may be used to plan future research and improve educational practice in public kindergartens.

## 1 Introduction

Modern individuals are experiencing increasing pressure from both normative and nonnormative stressful events (Schwarzer and Schulz, 2003). The amount and rate of information change is growing (Toffler, 1970), as is the number and frequency of natural, anthropogenic, and socio-economic threats, such as disasters, and military conflicts (Bennouna et al., 2020; Ivanov et al., 2022; Meshkova et al., 2022; UNDRR, 2020). In many countries, crime and violence rates remain quite high (Moen, 2019; Sánchez-Jáuregui et al., 2023). Together, these factors threaten the harmonious development and well-being of children, who are one of the most vulnerable groups in society (Khakimzyanov and Ryazanov, 2022). Children experience psychological impacts of stressful events—emotional reactions associated with the assessment and experience of what is happening. The experiences that children undergo in stressful events hinder the satisfaction of their basic needs for safety, predictability, connectedness, and development (Bowlby, 1979). Therefore, the emotional sphere is, on the one hand, the most sensitive to stressful events, and on the other, it is the main protective source, which emphasizes the non-direct environmental influences on child development and reactions. Environmental factors affect child development by the principle of refraction rather than by the principle of reflection: any environmental condition is refracted in a child’s mind in unique ways depending on his or her actual abilities and personal attitude to the situation (Gavrilova and Kornienko, 2025; Veresov, 2017).

## 2 Emotional intelligence, stress and resilience

Emotional intelligence is defined as an individual’s capacity to identify, use, comprehend, and control emotional information (Zhang et al., 2022). According to Trait Emotional Intelligence Model (Petrides, 2010), emotional intelligent contains four different large constructs: emotionality (emotion perception, expression, empathy, relationship, etc.), self-control (emotion regulation, stress management, adaptability, etc.), sociability (social competence, self-esteem, assertiveness, etc.), and well-being (happiness, optimism, etc.). Researchers have found that emotional intelligence serve a central function in psychological resilience (Armstrong et al., 2011; Maulding et al., 2012; Jowkar, 2007), appear to be crucial ability to successfully cope with challenge or misfortune (Guo et al., 2010; Sun et al., 2019; Wagnild and Young, 1993) and critical protective resource (Chen, 2012; Gong and Zhang, 2012). Emotional intelligence develops as children grow older. For instance, in infancy, individual differences in emotion regulation reflect brain-based temperamental factors, such as positive and negative emotionality, which may influence the quality of social interaction between child and caregiver. In children of preschool and elementary school age, language-based regulation of emotion becomes increasingly important. Increasing verbal capabilities allow the child to acquire rules for understanding and expressing emotion, such as “crying is okey” or “good girls do not behave rude,” and for communicating emotion to others (Zeidner et al., 2003). In adolescence cognitive and problem-solving strategies appear to cope with stress and challenges.

However, 5 the most intensive formation period for emotional intelligence is 3 to 7 years (Bozhovich, 1978; Zaporozhets, 1986). The emotional well-being of children at this age is highly sensitive to external factors. As a result, common reactions to stressful events include emotional symptoms such as anxiety, depression, fear, difficulties with emotion regulation, and behavioral problems (Fang et al., 2022; Luby et al., 2006; Platt et al., 2016; Zhang et al., 2022). Insufficiently formed emotional regulation, stress management, and adaptability, among other aspects of emotional intelligence, may not provide sufficient mental resilience.

Considering the dual role of emotional intelligence - on the one hand, heightened sensitivity to stressful factors, and on the other, a protective function - it is crucial to find ways to assist children by addressing negative emotional symptoms while also promoting emotional intelligence development and resilience.

## 3 The potential of play in overcoming negative emotional symptoms and improving emotional intelligence

The process of organizing psychological assistance for children must be based on knowledge of the psychological patterns of development. The patterns of child development have their own specificity at each age (Elkonin, 1971; Vygotsky, 1983; Zakharova and Machinskaya, 2023). Moreover, there are age differences in emotional coping with stress (Skinner and Zimmer-Gembeck, 2007). Together, this indicates the impossibility of creating a unified rehabilitation and preventive program for children of all ages. Therefore, in this study, we focused on finding ways of psychological preventive help and rehabilitation for young children aged 3 to 7 years, as the emotional development of children precisely in that period has the most significant impact on children’s future academic achievements and well-being (Currie and Almond, 2011; Bozhovich, 1978; Heckman, 2011; Zaporozhets, 1986).

Play is a leading activity between 3 and 7 years (Elkonin, 2005). The concept of ‘play’ can denote different types of child’s play activities. One of the most effective approaches to the theoretical analysis of play as a phenomenon is considered a cultural-historical approach (Vygotsky, 2017; Elkonin, 2005). Cultural-historical approach is based on the idea that child development takes place through the acquisition of historically created forms and modes of activity through communication rather than through the adaptation to the environment (Hedegaard and Fleer, 2008; Obukhova, 2012). According to Vygotsky (2017) play is a system of actions related to it. It is a culturally determined and most accessible activity for a child, arising spontaneously and bringing not only pleasure but also benefits for development (Fleer, 2022; Vygotsky, 2017; Zaporozhets and Elkonin, 1956). There are three most important characteristics of child’s play in relation to its role in a child’s psychological development: (1) in play the child creates and acts in an imaginary situation; (2) play is the source of psychological development and creates a zone of proximal development (ZPD) and, most importantly, (3) the development of play is characterized by changes in the relations between roles, rules and play actions (Veresov and Veraksa, 2023). Through play a child manifests their subjectivity and inner activity, endowing play with personal meaning and experiences while simultaneously acting as a subject of social action (Veraksa, 2011). In play, a child is usually focused on the process rather than the product (Veraksa et al., 2023). In play, the child learns what he cannot learn in daily life or education, for example, he masters the social relations of the adult world (Vygotsky, 2017).

From the point of view of the cultural-historical approach, there are four main types of play among children aged 3–7 years: the traditional ones are pretend play (or role play), play with rules, and play with toys (Elkonin, 2005), and recently appeared—digital play (Veraksa et al., 2023a; Veresov and Veraksa, 2023). In different types of play, a child gains fundamentally different experiences: from sharing toys with other players to emotional immersion in roles and enacting complex plots with peers, following complex rules. Additionally, the richness of play and child engagement in it may be influenced by the involvement of an adult who is a carrier of cultural experience and knowledge (Veresov et al., 2021).

In contemporary clinical and psychotherapeutic practice, therapeutic play, and in a broader context play therapy, have gained widespread recognition. Play therapy is defined as “a dynamic system of interpersonal relationships between a child and a therapist trained in play therapy procedures, who provides the child with play materials and facilitates the building of a safe relationship so that the child can fully express and explore their own self: feelings, thoughts, experiences, and behaviors, through play—the child’s natural means of communication” (Landreth, 1994). The goal of such therapy is not to change the child or teach them any specific behavioral skills, but to provide an opportunity to be themselves. In a safe play space, the child overcomes traumatic experiences through the symbolic expression of their feelings. Play therapy is used as a method of rehabilitation for children who have been subjected to abuse, serious traumatic events, developmental delays, and behavioral maladjustment, stressful events (LeBlanc and Ritchie, 2001). Research demonstrates the high effectiveness of play therapy and therapeutic play to reduce negative emotional symptoms such as anxiety, depression, fears and others (Baggerly, 2004; LeBlanc and Ritchie, 2001; Stulmaker and Ray, 2015).

Despite the effectiveness of play therapy and therapeutic play, there are a few limitations to these tools. The key constraint is the mandatory, lengthy, specialized training of the therapist. The therapist must be well-versed in both play and group therapy. A play therapist must sensitively understand and reflect the child’s emotional state during play. They must be fully engaged in the child’s play. The professional skill of a play therapist also includes their internal attitudes and ways of personal behavior, such as “the basic idea that the child is inherently motivated to grow and mature... And a deep and firm belief in the child’s ability to constructive self-direction” (Landreth, 1994). Secondly, play therapy and therapeutic play are long-term interventions. According to meta-analyses, the effects are observed after about 30 sessions, or 6 months of rehabilitation (LeBlanc and Ritchie, 2001). Together, this makes play therapy and therapeutic play difficult options for providing psychological help and prevention in public institutions. While parents rarely seek specialized help from psychotherapists for their young children.

To sum up, play is a space where a child realizes personal meanings and experiences, hones self-regulation skills and interactions with peers, and adults. Play has multifaceted influence on a child’s social–emotional development. Play therapy is actively used for addressing negative emotional symptoms in children. However, play therapy requires in-depth specialist training and long-term sessions, making its application in public institutions such as kindergartens difficult. Therefore, there is an important task to determine the effectiveness of play organized outside the therapeutic setting (e.g., therapeutic relationships, individual diagnosis, deep immersion of a specialist in the personal experiences of a child, compliance with therapeutic procedures, therapist training and supervision) to overcome negative emotional symptoms, develop emotional intelligence and stress resilience. For the purposes of this study, play organized outside of a therapeutic setting will be called a non-therapeutic play. Non-therapeutic play includes, for example, pretend play, play with rules, a digital play, free play with toys and other types of play. The spontaneous and child-controlled nature of non-therapeutic play indicates its potential for correcting and preventing emotional symptoms in children. However, this assumption needs confirmation.

## 4 Current study

One of the most effective approaches to the theoretical analysis of play as a phenomenon is the cultural-historical approach. It has a deep theoretical development of this topic and includes a set of practical tools for analyzing play as a phenomenon (Zaporozhets and Elkonin, 1956; Veraksa N. E. et al., 2022; Vygotsky, 2017). Therefore, the first goal of this review is to assess the possibilities of using play outside of a therapeutic context to reduce negative emotional symptoms such as anxiety, depression, fear, emotional-behavioral problems and develop emotional intelligence in children aged 3–7 years. The second goal is to analyze the features of play from the perspective of the cultural-historical approach, which provide its corrective, preventive, and developmental potential. The analysis of the features of the play in this work implies the identification of characteristics that ensure the effect as explained by the authors of the articles.

## 5 Methods

### 5.1 Search strategy

This systematic literature review was conducted to collect and analyze the most relevant empirical evidence on the impact of using play outside a therapeutic setting to reduce negative emotional symptoms in children aged 3–7 years. The PRISMA method was used for literature search and review to simplify the revision of the roadmap structure for research purposes (Moher et al., 2015). To this end, a literature search was carried out based on articles published in two databases: MEDLINE/PubMed and ScienceDirect. The selection of publications was carried out over a two-month period, up to and including 07/06/2024 and additionally for 3 weeks until 12/11/2024. To focus on the most current and relevant play practices, we analyzed papers published in the last 5 years (2019–2024). For the search strategy, 14 blocks of descriptors, combined using the Boolean operator AND, were utilized: (1) “play & “emotional intelligence”; (2) “play & stress”; (3) “play & anxiety”; (4) “play & depression”; (5) “play & emotional regulation”; (6) “play & fear” (7) “play & “emotional or behavioral problems” (8) “game & “emotional intelligence”; (9) “game & stress”; (10) “game & anxiety”; (11) “game & depression”; (12) “game & “emotional regulation”; (13) “game & fear” (14) “game & “emotional or behavioral problems.” In ScienceDirect, the descriptor “preschooler” was added to each block, as this database does not have the capability to automatically filter articles by a specific age group. In PubMed, the automatic selection option was used to find articles with sample ages of 2–5 and 6–12 years. In both databases the automatic selection option was used to find research articles.

### 5.2 Selection criteria

Articles had to meet the following criteria to be included in the study:

(1) Sample age: sample include normo-typic children between the ages of 3 and 7;
(2) Type of study: empirical studies analyzing the effects, relationships, or mechanisms of play;
(3) Type of study subject: using any type of play except therapeutic play to reduce the negative emotional symptoms or improve emotional intelligence;
(4) Statistical analysis: using qualitative or quantitative statistical tests to derive results.

The exclusion criteria for articles were as follows:

(1) Articles written in languages other than English;
(2) Articles with no access to their full texts;
(3) Withdrawn articles.

Despite the fact that these exclusion criteria are common to PRISMA reviews, they impose limitations on the article selection and narrow the scope of the studied works.

### 5.3 Study selection

First, the articles were screened based on their titles and abstracts. The selection process involved identifying studies related to the subject and eliminating those that did not meet the inclusion criteria or were unrelated. A complete analysis of the chosen studies was performed by critically reading the full texts. Any studies that did not meet the set criteria, as well as duplicate records, were excluded.

**Figure 1:**
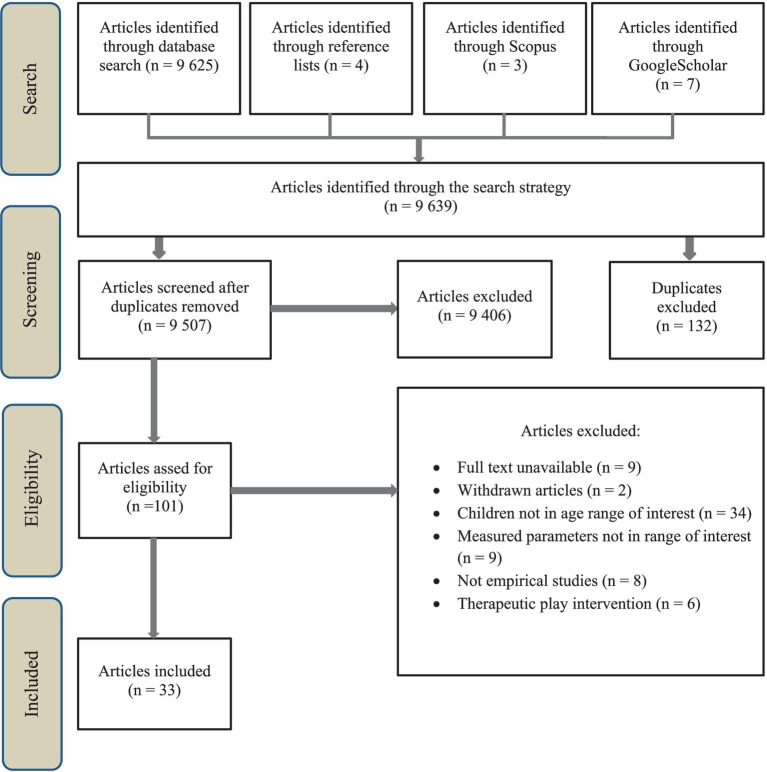
PRISMA—study flow diagram.

A total of 9,625 articles were identified through the initial database keyword searches. An additional 14 records were identified via Scopus, Google Scholar, and references of meta-analyses on similar subjects. Of these 9,639 articles, 132 duplicates were excluded. Screening for inclusion or exclusion criteria resulted in 101 studies being identified after reading their titles and abstracts. After a critical review of the full texts, 33 studies were included in the further qualitative review analysis (see Figure 1).

This review did not include an assessment of the methodological quality of the selected works because the main goal was to identify the potential and features of play outside a therapeutic setting for overcoming negative emotional states and to improve emotional resilience. To eliminate this limitation, a criterion on statistical data processing was included in the selection criteria, and the methodological characteristics of the selected studies were analyzed.

### 5.4 Categorization of play interventions

Types of play used in different play interventions were determined based on cultural-historical approach. For some interventions involving non-directive child activity, several categories have been identified. During the review, a new category “outdoor play” was also added, which emphasizes playing on the playgrounds. Outdoor play may include play with rules (for example, catch-up or hide-and-sick) or a pretend play on playground. However, the descriptions of the interventions were not sufficient to accurately attribute them to one of the initial categories. In addition, outdoor play also has its own unique characteristics, such as body movement, interaction with nature, and others.

The categorization was carried out by one expert.

## 6 Results

### 6.1 Description of included studies

The review included 33 empirical studies aimed at assessing the potential of play to overcome negative emotional symptoms and improve emotional intelligence outside the therapeutic setting. Tables 1, 2 provide detailed information of the included studies in the following areas: methodological characteristics of the included studies (Table 1), play intervention characteristics and features of play providing the effect (Table 2).

**Table 1:** Methodological characteristics of included studies.

Author, year	Target parameters	Methods and sample
Akhtar et al. (2023)	Anxiety	***Age***: 3–5 (M = 3.84) ***Size:*** 36 ***Design:*** Randomized control experiment ***Instruments:*** modified Yale Preoperative Anxiety Scale (mYPAS) (Annexure III)1 ***Statistical Tests:*** Student’s t-test
Arıkan and Esenay (2020)	Anxiety, fear	***Age***: 6–9 ***Size:*** 108 (50% males) ***Design:*** Randomized controlled experiment (3-parallel arms) ***Instruments:*** Visual Analog Scale, Wong-Baker FACES Pain Rating Scale, Children’s Fear Scale ***Statistical Tests:*** Student’s t-test, Chi-square
Azher et al. (2020)	Anxiety	***Age:*** 6–8 ***Size:*** 48 ***Design:*** Pretest-posttest quasi-experiment ***Instruments:*** Finger pulse oximeter, Venham’s anxiety and behavior rating scale ***Statistical Tests:*** Student’s t-test, Chi-square test
Bao et al. (2022)	Social anxiety	***Age:*** 4–7 ***Size:*** 87 ***Design:*** Correlational study ***Instruments:*** Woolley and Lowe assessment tool for play areas, Social Anxiety Scale for Children-Revised, Normalized difference vegetation index of the urban green spaces ***Statistical Tests:*** Spearman and Pearson correlation tests, stepwise regression analysis, ANOVA
Bargale et al. (2021)	Anxiety	***Age:*** 6–12 (M = 8.4) ***Size:*** 60 (48% males) ***Design:*** Pretest-posttest quasi-experiment ***Instruments:*** Animated emoji scale ***Statistical Tests:*** Student’s t-tests
Bauer et al. (2021)	Emotion comprehension, prosocial behavior	***Age***: 3–5 (M = 4.2) ***Size:*** 284 ***Design:*** Correlational study ***Instruments:*** Peabody Picture Vocabulary Test, NEPSY-II subtests, Childhood Imagination Questionnaire ***Statistical Tests:*** Structural Equation Modeling
Bawaeda et al. (2023)	Anxiety	***Age:*** 1–12 (M = 4.3) ***Size:*** 66 (56% males) ***Design:*** Randomized controlled experiment ***Instruments:*** Visual Facial Anxiety Scale ***Intervention:*** Pop-it therapeutic play ***Statistical Tests:*** Spearman correlation tests, Wilcoxon test, Mann–Whitney test
Chaurasia et al. (2019)	Anxiety	***Age:*** 4–8 (M = 4.75) ***Size:*** 80 (81% males) ***Design:*** Randomized controlled experiment ***Instruments:*** Modified-Yale Preoperative Anxiety Scale ***Statistical Tests:*** Student’s t-test, Mann–Whitney test
Dwairej et al. (2020)	Anxiety	***Age***: 5–11 (M = 6.5) ***Size:*** 128 (45.3% males) ***Design:*** Randomized controlled experiment ***Instruments:*** Modified-Yale Preoperative Anxiety Scale ***Statistical Tests:*** Student’s t-test
Goyel et al. (2022)	Anxiety	***Age***: 4–7 ***Size:*** 125 ***Design:*** Randomized controlled experiment (5-parallel arms) ***Instruments:*** Facial Anxiety Scale, pulse rate, partial pressure of oxygen, systolic blood pressure, diastolic blood pressure, salivary flow rate, salivary pH measurements ***Statistical Tests:*** One-way ANOVA, Student’s t-test, Kruskal–Wallis *H* test
Horoz et al. (2022)	Emotional and behavioral problems	***Age***: M = 6.02 toward the end of kindergarten ***Size:*** 731 ***Design:*** Randomized control experiment ***Instruments:*** Problem Behavior at School Interview (PBSI) ***Statistical Tests:*** latent growth curve / model
Islaeli et al. (2020)	Anxiety	***Age:*** 3–7 ***Size:*** 33 (42.4% males) ***Design:*** Pretest-posttest quasi-experiment ***Instruments:*** Faces Anxiety Scale for Children ***Statistical Tests:*** One Way ANOVA
Jaggy et al. (2023)	Emotional understanding, prosocial behavior, emotional regulation, cooperative behavior	***Age***: 2.5–5 (M = 3.6) ***Size:*** 211 (53.6% males) ***Design:*** Randomized controlled experiment ***Instruments:*** Reported social pretend play competence scale, Extended Theory-of-Mind Scale, Subtest social–emotional competence of the Intelligence and Developmental Scales – Preschool ***Statistical Tests:*** Repeated measures ANOVA
Jones et al. (2021)	Anxiety, fear	***Age:*** 5–10 (M = 6.68) ***Size:*** 50 (44% males) ***Design:*** Pretest-posttest quasi-experiment ***Instruments:*** Modified-Yale Preoperative Anxiety Scale ***Statistical Tests:*** Student’s t-tests, Wilcoxon test
Karaca and Guner (2022)	No significant effects	***Age***: 4–6 ***Size:*** 94 (48.3% males) ***Design:*** Randomized controlled experiment ***Instruments:*** Children’s Fear Scale, Children’s State Anxiety Scale, pulse rate, oxygen saturation, crying time ***Statistical Tests:*** Friedman test, *t*-test, the Mann–Whitney *U* test, Wilcoxon test, Repeated-measures ANOVA
Kırkan and Kahraman (2023)	Anxiety, fear	***Age:*** 3–8 ***Size:*** 84 (58.3% males) ***Design:*** Randomized controlled experiment ***Instruments:*** Children’s Fear Scale, Children’s Anxiety Meter–State ***Statistical Tests:*** Student *t-*test
Kumar et al. (2019)	Anxiety, stress, mood, cortisol	***Age:*** 5-15(M = 8.6) ***Size:*** 60 (55% males) ***Design:*** Randomized controlled experiment ***Instruments:*** State–Trait Anxiety Inventory for Children, Ottawa stress scale, Ottawa mood scale, cortisol measurement ***Statistical Tests:*** Student *t-*test
Lekhwani et al. (2023)	Anxiety	***Age***: 4–8 ***Size:*** 150 (53% males) ***Design:*** Pretest-posttest quasi-experiment (5-parallel arms) ***Instruments:*** Physiologic-Pulse Rate, Facial Image Scale, Venham’s Anxiety Scale ***Statistical Tests:*** Repeated-measures ANOVA, Friedman test
Leung et al. (2022)	Behavioral problems	***Age***: 3 (M = 3.5) ***Size:*** 267 (54.6% males) ***Design:*** Randomized controlled experiment ***Instruments***: Strength and Difficulties Questionnaire, Parent–Child Interaction Scale, Emotion-Related Parenting Styles, Parental Stress Scale questionnaires ***Statistical Tests:*** Student’s t-test
Matthyssens et al. (2020)	Anxiety	***Age***: 5–11 (M = 7.3) ***Size:*** 72 (68.1% males) ***Design:*** Randomized controlled experiment ***Instruments:*** Visual Analog Scale ***Statistical Tests:*** Linear mixed model
Maru et al. (2023)	Anxiety, pain	***Age***: 4–7 (M = 5.62) ***Size:*** 156 ***Design:*** Randomized control experiment ***Instruments:*** Wong-Baker Faces Rating Scale (WBFRS), measure of heart rate ***Statistical Tests:*** Student’s t-test, Chi-square test
Meng et al. (2022)	Emotional regulation	***Age:*** 4.5 ***Size:*** 1, female ***Design:*** Case study ***Instruments:*** Observation ***Statistical Tests:*** Qualitative data analysis
Nicolaidou et al. (2022)	Emotional regulation, anger	***Age***: 4–6 ***Size:*** 20 ***Design:*** Qualitative study ***Instruments:*** Semi-structured interview ***Statistical Tests:*** Qualitative data analysis
Petersen and Holodynski (2020)	No significant effects	***Age***: 3–6 (M = 5.1) ***Size:*** 52 (50% males) ***Design:*** Randomized controlled experiment ***Instruments:*** Disappointing gift task ***Statistical Tests:*** mixed ANOVA, Student’s t-test
Richard et al. (2021)	Emotion comprehension, aggressive bahavior	***Age***: 5–6 (M = 5.9) ***Size:*** 79 (54.4% males) ***Design:*** Randomized controlled experiment ***Instruments:*** The emotional label comprehension task, Test of Emotion Recognition, Contextual Task, Challenging situation task-Revised, Structured interview on negative emotion regulation strategies ***Statistical Tests:*** ANOVA
Richard et al. (2023)	Emotion comprehension, aggressive bahavior	***Age***: 5–6 (M = 5.75) ***Size:*** 180 (49.4% males) ***Design:*** Randomized controlled experiment ***Instruments:*** The emotional label comprehension task, Test of Emotion Recognition, Emotion comprehension task, Challenging Situation Task, Prosocial orientation, Strengths and Difficulties Questionnaire, Emotion Regulation Checklist, ***Statistical Tests:*** Student’s t-test, Mann–Whitney test, MANOVA
San et al. (2021)	Emotional intelligence, social and emotional development	***Age***: 3–5 ***Size:*** 90 ***Design:*** Randomized control experiment ***Instruments:*** Naing scale of social and emotional development for preschoolers ***Statistical Tests:*** Student’s t-test
Selzing-Musa et al. (2021)	Resilience skills, emotional regulation	***Age***: 5–7 ***Size:*** 40 ***Design:*** Non-equivalent control experiment ***Instruments:*** Emotional Skill Rating Scale (ESRS) ***Statistical Tests:*** ANCOVA
Shorer et al. (2021)	Emotional regulation, anxiety	***Age***: 2–8 (M = 5.16) ***Size:*** 137 ***Design:*** correlational study ***Instruments:*** The Parental Playfulness Questionnaire (PPQ), Revised Preschool Anxiety Scale (PAS-R), Emotions Questionnaire for parents ***Statistical Tests:*** two-tailed Pearson Correlations
Sobko et al. (2020)	Perceived stress, aggressive behavior, prosocial behavior, gut microbiota, fecal serotonin	***Age***: 2–5 (M = 3) ***Size:*** 45 (51.1% males) ***Design:*** Randomized controlled experiment ***Instruments:*** Perceived Stress Scale for Children, measures for microbiota and fecal serotonin ***Statistical Tests:*** Wilcoxon test, Spearman correlation, logistic regression
Stavrou (2019)	Emotional regulation, anger, dissatisfaction	***Age***: 4 ***Size:*** 1 (male) ***Design:*** Case study ***Instruments:*** Emotion Regulation Checklist, Delay Gratification Task, Task of Effortful Control ***Statistical Tests:*** Qualitative data analysis
Ünver et al. (2020)	Anxiety	***Age:*** 5–12 (M = 8.5) ***Size:*** 60 (72% males) ***Design:*** Randomized controlled experiment ***Instruments:*** Visual Facial Anxiety Scale, Facial Affective Scale ***Statistical Tests:*** Mann–Whitney test, Wilcoxon test
West et al. (2020)	Anxiety	***Age:*** 3–7 (M = 4.75) ***Size:*** 59 (59% males) ***Design:*** Randomized controlled experiment ***Instruments:*** Modified-Yale Preoperative Anxiety Scale ***Statistical Tests:*** ANCOVA, Wilcoxon test

**Table 2:** Play intervention characteristics.

Author, year	Play type	Duration	Special materials/conditions	Additional training of intervention providers	Features of play providing the effect
Akhtar et al. (2023)	Play with rules	1 play session, 60 min before medical procedure	Rewards (toy cars), balloons, toy medical equipment	X	Creation of a joyful, familiar, and predictable environment; incentives
Arıkan and Esenay (2020)	Adult-initiated play, play with a toy	1 play session, up to 60 min before medical procedure	Rotatable wooden toy	X	Distraction of the child’s attention
Azher et al. (2020)	Play with rules	Play session during dental procedures	Bubble balloons	Mini-guideline training for nurses or other assistants	Aerobic exercises and relaxation; distraction of the child’s attention.
Bao et al. (2022)	Outdoor play activities	–	Outdoor play spaces	X	Opportunities to practice outdoor activities (running, ball games etc.), which are aerobic exercises; positive social interaction
Bargale et al. (2021)	Play with rules	Play session during medical procedures	X	Mini-guideline training for nurses or other assistants	Aerobic exercises and relaxation; distraction of the child’s attention
Bauer et al. (2021)	Pretend play	–	X	X	Imaginary situation; enacting and re-enacting play roles; development of self-regulation
Bawaeda et al. (2023)	Play with a toy	Play session during medical procedures	Pop-it	X	Distraction of the child’s attention
Chaurasia et al. (2019)	Play with rules and medical equipment, Adult-involved play	1 play session before medical procedure	Incentives, medical equipment	X	Creation of joyful, familiar, and friendly environment; distraction of child’s attention; incentives; parental presence
Dwairej et al. (2020)	Digital play	1 play session before medical procedure	Digital device, medical equipment	NR	Distraction of the child’s attention
Goyel et al. (2022)	Play with rules, digital play, pretend play	Play session during medical procedures	Digital device	Mini-guideline training for nurses or other assistants	Creation of a joyful, familiar, and friendly environment; positive social interaction
Horoz et al. (2022)	Play with rules	Play session 3 times a week for 2 years, 15–60 min	Cards, incentives	Mini-guideline training for teachers or other assistants	Creation of a joyful, familiar, and predictable environment; social interaction; group responsibility; incentives
Islaeli et al. (2020)	Play with a toy	1 play session before medical procedure	Puzzles	X	Creation of a joyful, familiar, and friendly environment; relaxation; use of special material (puzzles)
Jaggy et al. (2023)	Pretend play	6 weekly group play sessions, 30-min	NR	X	Opportunity to experiment with social behavior; positive social interaction; creation of a safe environment for interpersonal communication
Jones et al. (2021)	Pretend play with medical equipment with, adult-involved play	1 play session before medical procedure, 20-min	Toy medical equipment	X	Positive social interaction; creation of a joyful, familiar, and friendly environment; expression of feelings; regaining a sense of control
Karaca and Guner (2022)	Adult-initiated play, play with a toy	1 play session before medical procedure	Music toy	X	No effect
Kırkan and Kahraman (2023)	Pretend play with medical equipment, free play	1 play session before medical procedure	Toy medical equipment (toy nebulizer, toy mask, and amigurumi doll)	X	Creation of a joyful, familiar, and friendly environment; expression of feelings and thoughts; regaining a sense of control
Kumar et al. (2019)	Pretend play with toys, free play with toys, free play	1 play session before medical procedure	Digital device, funny video, toys,	Parents’ preoperative counseling before surgery	Creation of a joyful, familiar, and friendly environment
Lekhwani et al. (2023)	Play with rules	Play session during dental procedures	X	Mini-guideline training for nurses or other assistants	Creation of a joyful, familiar, and friendly environment
Leung et al. (2022)	Pretend play, adult-involved play	NR	NR	20 weekly educational sessions for parents, 1.5-h	Emotional scaffolding; positive parent–child interaction
Matthyssens et al. (2020)	Digital play	1 play session before medical procedure	Digital device, CliniPup app	X	Familiarization with information about the procedure; involvement
Maru et al. (2023)	Digital play	3 times a day for 1 week	Digital device, Tiny Dentist app	X	Creation of a joyful, familiar, and friendly environment
Meng et al. (2022)	Pretend play	10 weeks group play session	NR	X	Enacting and re-enacting play roles; facilitation of the interaction between raw emotions and conscious feeling state
Nicolaidou et al. (2022)	Digital play	1 play session	Digital device, special app	X	Involvement; educational component
Petersen and Holodynski (2020)	Pretend play	Group pretend play (being magicians) during the task	Play material	X	No effect
Richard et al. (2021)	Pretend play	11 weekly play sessions, 60 min	NR	20 h of specific training for teachers	Emotional scaffolding; creation of a safe environment for interpersonal communication; collaborative nature of conversations; positive social interactions
Richard et al. (2023)	Pretend play	11 weekly play sessions, 60 min	NR	20 h of specific training for teachers	Imaginary situation; enacting and re-enacting play roles; opportunity to experiment with social behavior and emotions; repetition of emotions; verbalization; educational component
San et al. (2021)	Pretend play with toys, free play with toys, adult-involved play	NR	NR	Trainings for caregivers	Positive parent–child interaction; a material-rich environment; educational component
Selzing-Musa et al. (2021)	Pretend play	6 weeks daily play sessions	Classrooms arranged into learning centers, toys to play transportation, petty trading, cooking, building and farming	X	Imaginary situation; gaining initiative and creativity; enacting and re-enacting play roles; open-ended perspectives; expression of emotions and thoughts
Shorer et al. (2021)	Adult-involved play	–	Playing with parents	X	Positive parent–child interaction; involvement of adult as a socialization agent
Sobko et al. (2020)	Outdoor play activities, play with rules	10-week daily sessions of Play&Grow program	Outdoor play spaces	Parents’ counseling	Exposure to the natural environment; expression of feelings; relaxation exercises
Stavrou (2019)	Pretend play	3 months of weekly play sessions, 10-min	Lego, play structure protocol	Mini-guideline training for teachers, parents or other assistants.	Imaginary situation; communication through nonverbal, symbolic means
Ünver et al. (2020)	Play with rules	1 group play session, up to 30 min before medical procedure	Jenga™ game	X	Distraction of the child’s attention; creation of a joyful atmosphere
West et al. (2020)	Pretend play with medical equipment, free play	1 play session before medical procedure, 15-min	Child Life program, toy medical equipment	Mini-guideline training for nurses or other assistants.	Creation of joyful, familiar, and friendly environment

#### 6.1.1 Study design

Three of the selected works are qualitative studies (Nicolaidou et al., 2022; Meng et al., 2022; Stavrou, 2019), such as case studies, which do not aim to identify the effects of play on the mental development of preschoolers but focus specifically on identifying the features of play providing the impact on the child’s psyche. Among the quantitative studies, the most common experimental design is the randomized controlled experiment - 23 studies, which indicates a relatively high reliability of the obtained results and conclusions. Four studies are quasi-experiments comparing the effectiveness of different types of play and other interventions (Azher et al., 2020; Bargale et al., 2021; Islaeli et al., 2020; Jones et al., 2021). However, the data on the effectiveness of the analyzed types of play and the reliability of the obtained conclusions should be considered with the relativity of the results in mind for these studies. Three included studies provide information on the relationship between play activities and indicators of child emotional development (Bao et al., 2022; Bauer et al., 2021; Shorer et al., 2021). None of the studies included a follow-up post-test, which imposes limitations on conclusions about the sustainability of the results obtained.

#### 6.1.2 Sample characteristics

The average sample size of the selected studies, excluding qualitative ones, is 122.4 participants, indicating the scale of the selected studies. Thus, the results of the analyzed studies can be considered reasonably reliable. The mean age of participants in the reviewed studies is 5.3 years.

#### 6.1.3 Play intervention characteristics

The selected studies showed the effectiveness of the following types of play: pretend play (*n* = 15), play with rules (*n* = 9), play with toys (*n* = 9), digital play (*n* = 5), adult-involved play (*n* = 4), adult-initiated play (*n* = 3), outdoor play (*n* = 2). Let us clarify that adult-involved play implies his participation as a respectful observer or player, whereas adult-initiated play implies attracting attention of the child, trying to actively involve a child in the game. One intervention can include several types of play, for example, the nurse involves a child to play with a doctor-doll (adult-initiated play + play with toys).

The duration of play interventions varies from one 20-min session to three times a week 15–60-min sessions for 2 years. 18 of 33 studies assessed one-time play sessions, while 15 studies assessed long-term implementation of play. 24 of the proposed play interventions assume the use of special materials (medical toy equipment, electronic devices, bubble balloons, etc.) or availability of special conditions (outdoor play spaces, parent involvement, etc.). However, all the necessary materials are readily available.13 of 33 play interventions involve special training of teachers, parents, nurses, and other assistants. The minimum duration of training is 1 h, the maximum is 20 h.

18 of 33 studies evaluated play in the medical context such as staying at hospitals or dental offices, in conditions of necessary medical interventions, surgeries, or in the presence of medical diagnoses. In one study, play was used to correct the emotional sphere of a child in an orphanage (Meng et al., 2022).

Table 2 provides more detailed description of play intervention characteristics.

#### 6.1.4 Effects of play interventions

From a substantive perspective, the results of the selected studies demonstrate the potential and effectiveness of play in: reducing anxiety (*n* = 18); developing emotional intelligence and its components (*n* = 11); declining emotional-behavioral difficulties (*n* = 8); overcoming fear (*n* = 3); reducing stress levels (*n* = 2). However, 2 studies did not find a significant effect of play, particularly adult-initiated play with toys and pretend play, on the mental state of young children (Karaca and Guner, 2022; Petersen and Holodynski, 2020).

Results indicated that is no clear correlation between the type of play used and its impact on a specific psychological parameter. One type of play in different studies may show effectiveness concerning various psychological indicators. Detailed information about the impact of different types of play on child mental state and development according to the selected studies is present in Table 3.

**Table 3:** Effects of play interventions on child mental state and development.

Play type/ psychological parameters	Anxiety	Stress	Fear	Mood	Subjective well-being	Emotional intelligence and its components	Aggressive behavior	Emotional-behavioral problems
Pretend play	+	+	+	+	+	+	+	+
Play with rules	+	+	+					+
Digital play	+	+		+		+		
Outdoor play	+	+					+	+
Adult-involved play	+		+			+		+
Adult-initiated play	+		+			+		
Play with toys	+							

#### 6.1.5 Features of play providing the effect

As a result of the qualitative analysis of the selected studies, 28 features through which play impacts the mental state of young children were identified. In accordance with the cultural-historical understanding of the nature of play, we identified 7 key generalized blocks of mechanisms:

1) Dialectical structure of play (imaginary situation, communication through nonverbal, symbolic means, enacting and re-enacting play roles, development of self-regulation, open-ended perspectives of play).
2) Emotional engagement and reflection (expression of feelings and/or thoughts, emotional scaffolding, facilitation of the interaction between raw emotions and conscious feeling state, repetition of emotions, verbalization).
3) Support of inner activity (regaining a sense of control and/or success, gaining initiative and creation).
4) Social interaction (positive social interaction, parental presence, positive child–parent interaction, opportunity to experiment with social behavior, collaborative nature of conversations, involvement of adult as a socialization agent, group responsibility).
5) Creation of a joyful, familiar, predictable and friendly environment (creation of a joyful, familiar, predictable, and friendly, creation of safe environment for interpersonal communication).
6) Active involvement (distraction of the child’s attention, involvement, incentives).
7) Opportunity to include additional information and/or techniques in play (relaxation exercises and/or techniques, familiarization with information about something / educational component included in play, aerobic exercises, exposure to the natural environment, use of special material).

## 7 Discussion

The primary goal of this review was to analyze the potential of play for improving emotional sphere in children aged 3–7 years outside of a psychotherapeutic setting, particularly its potential to reduce negative emotional symptoms and develop emotional intelligence. A total of 33 studies were analyzed in this review. Existing research indicates the potential of play for addressing the mental state of preschoolers, including anxiety, stress, emotional regulation, and emotional-behavioral difficulties. Seven key pathways through which play affects children’s mental state were identified. However, there is no empirical evidence for the sustainability of these effects.

The analysis reveals four trends in studying play in preschool-aged children and its application possibilities. The first trend indicates that play can be effectively applied to develop or address a wide range of psychological parameters. The second trend shows that play can have a comprehensive impact on the mental state of children through various pathways affecting motivational, emotional, cognitive, and behavioral levels. The third trend descripts different types of play and reflects the significance of pretend play for the emotional development of young children, as it is the most frequently suggested intervention to reduce negative emotional symptoms and to improve emotional intelligence and its components. Play with rules, play with toys and digital play have also demonstrated their effectiveness in various studies. The fourth trend concerns the prevalence of clinical studies on the use of play to reduce negative mental states in children undergoing medical procedures compared to other stressful events.

### 7.1 The potential of play outside of a therapeutic setting to reduce emotional symptoms and improve emotional intelligence

The analysis of the selected studies revealed that play has a multifaceted impact on the mental state of preschoolers. Specifically, the review identified 13 psychological parameters in the emotional and social domains of the child that can be developed or corrected through play. Studies have shown the effectiveness of play in addressing all major psychological consequences of stressful events. Play not only helps relieve tension, reduce stress and anxiety but also fosters the development of emotional regulation and comprehension (Meng et al., 2022; Selzing-Musa et al., 2021), which contributes to stress resilience and coping with challenges (Chen, 2012; Gong and Zhang, 2012; Guo et al., 2010). The possibility of receiving support for subjectivity in the play and sufficient autonomy helps not only to overcome negative feelings but also develop self-help skills for other stressful events.

It is important to emphasize that the analysis of the studies demonstrated the accessibility of using play. Unlike therapeutic play and play therapy, organizing non-therapeutic play does not require extensive additional training for specialists. Non-therapeutic types of play and programs based on them can be implemented either without specialized training for educators, nurses, psychologists, and other professionals who work with children or with a brief introduction to instructions, game structure, and preparation, as well as training for parents. But we should note that organizing even non-therapeutic play requires basic psychological or pedagogic education, knowledge about the patterns of child mental development in different ages. Such types of play as pretend play, play with rules and toys, digital play, outdoor play can be organized both at home and in kindergartens.

A special role in using play to overcome negative emotional states and develop emotional intelligence belongs to toys. It is important that toys and play materials (e.g., costumes and props) correspond to the themes of the child’s experiences and are sufficiently varied (Goldstein, 1994). For instance, to address anxiety and fears related to adapting to a new kindergarten, it is necessary to have a sufficient number of toys that can represent children and educators in the play (Gorshkova and Lvova, 2023). At the same time, the main advantage of play is opportunity to use symbolic toys and open play materials (ribbons, fabrics, sticks, etc.), which facilitates the task of selecting toys (Veraksa A. N. et al., 2022).

Finally, the studies reviewed demonstrated effectiveness of play in different timeframes. One-time play sessions are commonly used to reduce situational emotional symptoms (Akhtar et al., 2023; Jones et al., 2021). Systematic play sessions contribute both to the negative symptoms reduction and emotional development (Jaggy et al., 2023; Stavrou, 2019). Prolonged non-therapeutic play exposure leads to an effect after only 6 weekly sessions. This indicates the potential of using non-therapeutic play in educational practice.

Thus, play outside a therapeutic setting can be used for overcoming negative emotional symptoms and develop emotional intelligence. In the reviewed studies, play outside the therapeutic context was used in education, parenting practices, before medical procedures, and to adapt to an orphanage. At the same time, the accessibility of its organization, corrective and developmental potential indicates the possibility of its use for children faced with various stressful situations. However, this assumption needs further research.

### 7.2 Features of different play types providing the effect

Play can have a comprehensive impact on the mental state of children through various pathways affecting motivational, emotional, cognitive, and behavioral levels. Seven blocks of identified pathways can be used separately or simultaneously, increasing the development potential of the play. Different types of play have different characteristics and combine different pathways of influence on the child emotional sphere.

In contemporary preschool education, significant importance is placed on pretend play as a special type of children’s activity, which is reflected in the number of studies where this type of play is used for development or correction. From the perspective of the cultural-historical approach, pretend play has the most essential impact on psychological development during preschool years (Hakkarainen and Bredikyte, 2010). Pretend play is a culturally conditioned type of activity where children, through imaginary situations, reproduce various aspects of adult life, thus learning social roles and relationships by enacting different plots (Elkonin, 2005). The uniqueness of pretend play lies in the emergence of a special relationship between the child and the situation, characterized by the dual nature of children’s experiences and actions (Veraksa, 2011; Vygotsky, 2017). In pretend play, the child operates on two levels—perceptual and symbolic—which together form the dialectical structure of play (Veraksa, 2022). For example, when a child builds a house out of pillows in play, they consider the real properties of the pillows but imagine them as a roof or bricks. While playing “hospital,” the child simultaneously cries like a patient and rejoices as a player. In other words, the child progresses to an activity centered on social relations and guided by an internal plan, leaving behind the play centered on and dominated by objects. Vygotsky conceptualizes this transition as a progressive separation between the visual field and the field of meaning (Hakkarainen and Brėdikytė, 2015; Hakkarainen et al., 2017; Vygotsky, 1978).

Therefore, according to the cultural-historical approach, pretend play serves as zone of proximal development for the child: what is challenging for the child to achieve through direct adult instruction becomes attainable in play, such as controlling aggressive behavior and regulating emotions (Vygotsky, 2017). A similar view on the developmental potential of pretend play can be found in other approaches. For example, Winnicott (1951) considered play as a transitional space because children use both objects and phenomena form the external world (e.g., toys) and aspects derived from their inner world (dreams or fantasies). In the transitional space, the child experiences his “omnipotence,” builds trust to the world, gets a new experience, and takes the next step toward development (Sagan, 2011). Additionally, in pretend play, the child reproduces models of social relationships (Elkonin, 2005). The child takes on various social roles, becoming an obedient student, a teacher, a hero, etc., and gains experience in partnership relationships with playmates. In play children exercises shared regulation, including emotional regulation, toward others because children must regulate their own behavior and, at the same time, control the behavior of playmates (Brėdikytė and Hakkarainen, 2017). These features of pretend play determine the variety of pathways impacting the child’s psyche. It is noteworthy that the review of studies showed that all 7 generalized blocks of mechanisms are characteristic of pretend play, which indicates its potential to address negative emotional symptoms and develop emotional intelligence in young children.

The review also showed that play with rules can contribute to reducing emotional symptoms in children. Play with rules is a type of play that imposes external rules that must be followed throughout the play (Veraksa et al., 2023). The main advantage of this type of play is that it allows to vary the complexity of the tasks for emotional regulation by adding new game components or exercises, for instance relax techniques or aerobic exercises (Azher et al., 2020; Bargale et al., 2021). In play with rules, through the addition of various components, an adult can transmit to a child cultural way of mastering their own internal processes. Following the rules of the game promotes the development of self-regulation, including emotional and social self-regulation (Savina and Wan, 2017). However, research showed that play with rules is effective for the development of older preschool children (6–7 years), while for younger preschoolers, following external rules remains a difficult task (Elkonin, 2005). For children under the age of 5–6 years, it is easier to follow the rules related to their self-chosen role in the pretend play. As well as pretend play, play with rules unfolds in imaginary situation, but commonly there is no plot and role development in play with rules, children do not use symbolic means. When playing with rules, children are more focused on following the rules or achieving a goal than on expressing their own emotions and experiences. To sum up, mentioned features limit the range of pathways through which play with rules can influence the child mental state and development. Main developmental mechanisms of play with rules are active involvement, opportunity to include additional information and/or techniques, imaginary situation.

It was revealed that play with toys can reduce negative emotional symptoms in children. The effect of playing with toys on a child mental state depends on the degree to which the child is immersed in an imaginary situation in play. When a child simply manipulates a toy, the effect either does not reach (Karaca and Guner, 2022) or through distracting the child’s attention helps to reduce anxiety (Arıkan and Esenay, 2020; Islaeli et al., 2020). In studies where a child creates a plot in a game with toys, indeed playing a pretend play, not only negative symptoms are reduced, but emotional regulation also develops (San et al., 2021). Thus, the number of pathways impacting the child’s mental state and development in play with toys depends on the quality and level of the certain game.

Digital play in the reviewed studies is also considered as a beneficial activity. Digital games are programs that organize and direct the play process on various electronic devices. In most studies digital play is used as a distractor or to introduce new information to a child (Dwairej et al., 2020; Matthyssens et al., 2020), which reduces anxiety, stress or fear. In one study digital play based on fairy-tale was proved to develop emotional regulation (Nicolaidou et al., 2022). However, first disadvantage of using digital play is social isolation while playing. Most digital games do not involve interaction with an adult or a peer, while positive social interaction is an important pathway to reduce negative emotional symptoms and develop emotional intelligence (Fonseca et al., 2021; Lozada et al., 2014; Smith and Carlson, 1997). Secondly, research have revealed that positive impact on child development from digital play is unsustainable (Veraksa et al., 2023b). Digital play does not contribute to systematic, qualitative changes in child development. Although some research uses digital play to address emotional symptoms in children, its corrective and developmental potential is limited.

The review of studies showed that outdoor play is another type of play to lessen emotional symptoms and improve emotional resilience. In the reviewed studies outdoor play implies the child’s play on the playground (Bao et al., 2022; Sobko et al., 2020). Thus, it may include elements from pretend play, play with rules or toys. In research it is highlighted that outdoor play commonly include physical activity (running, jumping, physical exercises, etc.), which helps to reduce tension (Wickel and Belton, 2016). Exposure to the natural environment also serves to overcome negative emotions (Sobko et al., 2020). Another, important mechanism in outdoor play is intensive social interaction with peers, which leads to socio-emotional development (Little and Wyver, 2008). Despite the mentioned pathways impacting the child’s mental state and development in outdoor play, its effectiveness depends on the specific child activity in it.

To sum up, the reviewed studies report that different types of play improve the emotional sphere of children. Pretend play involves the greatest number of pathways through which play influence the child psyche. At the same time, it is important to clarify that the corrective and developmental effect in non-therapeutic play is achieved not only through the play itself. Some features of the play providing the impact (e.g., emotional scaffolding, verbalization of emotions, parental presence, positive child–parent interaction, incentives, use of special material and additional information) depend on the involvement of a sensitive adult in the play. As a bearer of cultural experience and knowledge, the adult can enrich the child’s play, influence its depth and development, and provide models of actions, roles, and plots (Fleer, 2022). The participation of an adult in play ensures that the child gains unique experiences, thereby potentially determining the developmental potential of the play (Veresov et al., 2021). Thus, when implementing non-therapeutic play, it is important to consider not only the type of play and its features providing the effect but also the quality of adult involvement.

### 7.3 Review limitations

The main limitation of this review relate to the selection of publications. The selection process from search databases was conducted mainly without the use of automated algorithms. The search based on selected keywords might have excluded relevant studies, which reduced the number of reviewed studies. Additionally, this review did not include an assessment of the methodological quality of the studies using specialized tools (e.g., NHMRC framework, QualSyst, etc.). However, to eliminate this limitation methodological characteristics and statistical tests were analyzed in the review process.

### 7.4 Practical implications and future work

The following summarizes our findings about implementation of non-therapeutic play to reduce emotional symptoms and develop emotional intelligence in young children.

1. To reduce emotional symptoms and develop emotional intelligence in non-therapeutic play, several blocks of pathways identified in this review should be combined to ensure correctional and developmental potential. Primary attention should be paid to creating dialectical structure of the play creating an imaginary situation, as well as providing emotional scaffolding, supporting the child’s inner activity, and ensuring group interaction between children (Bauer et al., 2021; Landreth, 1994; Vygotsky, 2017).
2. Involving a sensitive adult (parents, teachers or other assistance) in the game as an equal player increases correctional and developmental potential of intervention (Jones et al., 2021; Leung et al., 2022; Veresov et al., 2021).
3. To reduce situational emotional symptoms one-time play sessions in an unfamiliar environment up to 60 min can be organized. The play should include familiarization with a new environment, for example, the use of new objects in a play with rules, the use of a new space for pretend play, mastering new information in a digital play (Akhtar et al., 2023; Nicolaidou et al., 2022).
4. To foster emotional development systematic play sessions can be organized at least weekly for 6 weeks (*in the reviewed studies 6 weeks of play sessions were the minimum option that had a positive effect*). Play sessions should include usage of symbolic means, enacting and re-enacting play roles, emotional engagement and reflection, support of inner activity, positive social interaction (Jaggy et al., 2023; Selzing-Musa et al., 2021).
5. Symbolic toys, changeable toys (e.g., Lego), and improvised materials (ribbons, boxes, sticks, leaves, fabric of different colors and sizes, plastic tableware etc.) can be used to organize the game, which will enhance emotional engagement and support an imaginary situation (Kırkan and Kahraman, 2023; Selzing-Musa et al., 2021; Stavrou, 2019).

A future study should focus on a narrower topic to undertake a comprehensive analysis, especially the corrective effect of non-therapeutic play in children with high level of emotional or behavioral symptoms. An alternative approach would include evaluating the implementation of non-therapeutic play for children faced with different stressful events other than medical procedures (e.g., relocation, family difficulties). Finally, it is necessary to conduct research to determine whether non-therapeutic play have a long-lasting sustainable effect.

## 8 Conclusion

This review analyzed 33 studies published in the last 5 years. It was shown that play outside the therapeutic setting holds significant emotional corrective and developmental potential in early childhood. According to the cultural-historical approach, 7 generalized blocks of play features providing the impact on children’s mental state and development were identified: dialectical structure of play, emotional engagement and reflection, support of inner activity, social interaction, creation of a joyful, familiar, predictable, and friendly environment, active involvement, and the opportunity to include additional information and/or techniques in play. Among the different types of play, pretend play showed the greatest potential. However, it is important to consider the benefits of other types of play to engage the maximum number of mechanisms impacting the child’s psyche and to maximize its effectiveness. The variety of pathways of impact and accessibility for implementation indicate the potential for organizing psychological support programs for children, and particularly for children facing with stressful events, based on play. The results of this work can be used for practical purposes, such as developing psychological support programs based on non-therapeutic play activities.

## Data Availability

The original contributions presented in the study are included in the article/supplementary material, further inquiries can be directed to the corresponding author.
